# Metabolomic Analysis of the *Takifugu Obscurus* Gill under Acute Hypoxic Stress

**DOI:** 10.3390/ani12192611

**Published:** 2022-09-29

**Authors:** Huakun Zhang, Ziwen Hu, Run Li, Yaohui Wang, Jinxu Zhou, Hao Xu, Guan Wang, Xuemei Qiu, Xiuli Wang

**Affiliations:** 1 College of Fisheries and Life Science, Dalian Ocean University, Dalian 116023, China; 2 Key Laboratory of Pufferfish Breeding and Culture in Liaoning Province, Dalian Ocean University, Dalian 116023, China; 3 Jiangsu Zhongyang Group Company Limited, Nantong 226600, China

**Keywords:** *Takifugu obscurus*, acute hypoxic stress, metabolomics, gill

## Abstract

**Simple Summary:**

*Takifugu obscurus* is an economically important aquaculture species in China. In recent years, the development of the domestic breeding industry of the globefish has been very rapid. However, oxygen fluctuations and nourishing substances in the aquaculture water have caused oxygen deprivation, which makes great economic losses in high-density farming. As the main respiratory organ of fish, gills are greatly affected by changes in dissolved oxygen. Therefore, in this study, we explored the molecular mechanism of hypoxia tolerance of pufferfish by analyzing the changes of metabolites in gill tissue under acute hypoxia. These data provide a scientific basis for the control of dissolved oxygen in the aquatic environment of *T. obscurus*, and also provide a reference for the breeding of the new varieties with low oxygen tolerance.

**Abstract:**

*Takifugu obscurus* has relatively small gills and gill pores. Consequently, a relatively low respiratory capacity. This fish is thus easily negatively affected by the low levels of dissolved oxygen (DO) that are common in high-intensity aquaculture. In order to clarify the mechanisms underlying the hypoxia response of *T. obscurus,* we used liquid mass spectrometry (LC–MS) to identify and quantify the metabolites present in the *T. obscurus* gill under the following conditions: normoxia (DO, 7.0 ± 0.2 mg/L), hypoxia (DO, 0.9 ± 0.2 mg/L), and reoxygenation (4, 12, and 24 h after return to normoxia conditions). We identified a total of 821 and 383 metabolites in the gill in positive and negative ion modes, respectively. Of the metabolites identified in positive ion mode, 136 were differentially abundant between hypoxia and all other conditions; of the metabolites identified in negative ion mode, 34 were differentially abundant between hypoxia and all other conditions. The metabolites which were differentially abundant under hypoxia primarily included glycerol phospholipids, fatty acids, hormones, and amino acids as well as related compounds. The pathways which were significantly enriched in the differentially abundant metabolites included the lipid metabolism, amino acid metabolism, purine metabolism, FoxO signaling pathway, and mTOR signaling pathway. Our results help to clarify the mechanisms underlying hypoxia tolerance and to identify hypoxia-related metabolites, as well as to highlight potential research targets for the development of hypoxic-tolerant strains in the future.

## 1. Introduction

Dissolved oxygen, which is necessary for aquatic animal survival and affects growth, metabolism, and other life activities, is a major limiting factor in aquaculture [1]. The concentration of dissolved oxygen in water generally depends on a number of factors, including biotic and abiotic factors. Under natural conditions, salinity, water temperature, surface runoff, circadian rhythms, and seasonal alternations can also result in dramatic fluctuations in dissolved oxygen levels [2]. Due to increases in water eutrophication over recent decades, global and seasonal hypoxic events have become increasingly common worldwide.

In general, fish feed normally in water with dissolved oxygen contents above 5 mg/L; when dissolved oxygen levels decrease below 2 mg/L, fish feed intake decreases significantly. At dissolved oxygen levels below 1 mg/L, fish exhibit “floating head” syndrome and may even asphyxiate [3]. Although fish have some ability to adapt to changes in dissolved oxygen, both short-term acute and long-term chronic hypoxia can affect a variety of physiological and biochemical processes, disrupting embryonic development, growth, and reproduction; modulating cellular tissue morphology and structure; and altering swimming and feeding behaviors [4,5]. For example, the growth rate of juvenile Atlantic sturgeon (*Acipenser oxyrinchus*) in hypoxic environments was about three times lower than growth rates in normoxic environments [6]. In addition, the ovaries of zebrafish (*Barchydanio rerio var*) under long-term hypoxia developed more slowly and produced fewer oocytes than the ovaries of normoxic zebrafish [7], while carp under hypoxic stress exhibited significantly decreased fertilization and hatching rates compared to normoxic carp [8]. Finally, after a month-long exposure to hypoxia (dissolved oxygen, 1.34 mg/L), androgen and estrogen levels in the killifish *Fundulus grandis* decreased significantly, with consequent decreases in reproductive ability [9]. These previous studies show that low levels of dissolved oxygen strongly affect growth and reproduction in a variety of fish, with serious implications for the development of aquaculture industry.

The pufferfish *Takifugu obscurus* (superclass Osteichthyes, order Plecoptera, family Tetraodontiformes) is a warm-temperate demersal migratory fish that is mainly distributed in benthic and pelagic waters offshore of China and in the middle and lower reaches of the Yangtze River. *T. obscurus* is more susceptible to low dissolved oxygen levels than other fish due to its weak respiratory capacity and thus has a low tolerance of hypoxia [1]. Cultivated *T. obscurus* require more than 6 mg/L dissolved oxygen, and hypoxic conditions caused by continuous rainy weather and high-density aquaculture will result in mass die-offs; the high dissolved oxygen requirements of this species, even at rest, are a result of its sexual ferocity, gluttony, high protein consumption, and elevated activity levels [10]. As aquaculture has also increased in popularity over recent years, fish quantities and densities have increased, with bait casting and relatively closed water bodies becoming more common. These factors have rendered hypoxic conditions more likely, strongly restricting the growth of the *T. obscurus* aquaculture industry. In order to improve the prospects of *T. obscurus* aquaculture, it is first necessary to understand the mechanisms underlying hypoxic stress in this species.

The fish gill is an organ essential to respiration and aquatic gas exchange that also plays important roles in osmoregulation, detoxification, immunity, and neuronal signaling. Because the gill is the first tissue to be affected by elevated temperatures and hypoxic conditions, this organ is a critical factor in the adaptation of fish to their environment [11]. Li et al. [12] have studied the gene regulation mechanism of dark streaks under hypoxic stress at the transcriptome level, but no metabolomics report has been investigated. We speculate that the metabolites in the gills of *T. obscurus* undergo some physiological changes under acute hypoxic stress to counteract the effects of the adverse external environment on the organism. Therefore, we used liquid chromatography–mass spectrometry (LC–MS) to identify the metabolites in the gill tissue of *T. obscurus* which were differentially abundant under hypoxic stress. Our results not only help to clarify the mechanisms of the hypoxia response in fish, but also provide reference data for the selection and breeding of new hypoxia-tolerant varieties.

## 2. Materials and Methods

### 2.1. Ethics Statement

All fish in this study were handled in strict accordance with Chinese legislation on scientific procedures on living animals. The protocol was approved by the ethics committee at Dalian Ocean University. Before sacrificing and handling, experimental fish were anesthetized with 100 ng/mL of ethyl 3-aminobenzoate methanesulfonic acid (MS-222, Sigma, St. Louis, MO, USA), and all efforts were made to minimize suffering. The field studies did not involve endangered or protected species.

### 2.2. Experimental Instruments and Reagents

The following instruments were used in this study: benchtop centrifuge TGl-16B (Shanghai Anting Scientific Instruments Factory, Shanghai, China), precision electronic balance JJ500 (Changshu Shuangjie Testing Instruments Factory, Suzhou, China), mass spectrometer Q ExactiveTMHF-X (Thermo Fisher, Waltham, MA, USA), chromatograph Vanquish UHPLC (Thermo Fisher, Waltham, MA, USA), chromatographic column Hypesil Gold column (100 mm × 2.1 mm, 1.9 μm; Thermo Fisher, Waltham, MA, USA), and cryogenic centrifuge D3024R (Scilogex, Rocky Hill, CT, USA). The following experimental reagents were used in this study: LC–MS grade methanol (MeOH; Thermo Fisher, Waltham, MA, USA), LC–MS grade formic acid (Thermo Fisher, Waltham, MA, USA), LC–MS grade ammonium acetate (Thermo Fisher, Waltham, MA, USA), and LC–MS grade water (Merck, Darmstadt, Germany).

### 2.3. Experimental Fish

We randomly selected 300 healthy *T. obscurus* (mean weight, 91.6 ± 22.9 g) for study from the *T. obscurus* breeding farm, Jiangsu Zhongyang Co. (Hai’an, Nantong, Jiangsu, China). Fish were transferred to experimental pools (water temperature, 24 ± 1 °C) and allowed to acclimate for one week. During the acclimation period, the dissolved oxygen level in the experimental pools was maintained at 7.0 ± 0.2 mg/L by adjusting the aeration, and fish were fed with compound feed and small fry. Fish were starved for 24 h before experimentation and were not fed during the experiment.

### 2.4. Experimental Design and Sample Collection

From the experimental pools of 300 fish, 120 fish were selected for the pre-experiment and the threshold of hypoxia was determined as 0.9 ± 0.2 mg/L (at this dissolved oxygen value, the fish reach the limit of hypoxic tolerance and lose equilibrium). In the formal experiment, 40 fish were randomly selected and transferred to a 100 L tank (water temperature, 24 ± 1 °C). The experimental tank was tightly sealed, and the rapid decrease in dissolved oxygen content was monitored in real time. After dissolved oxygen levels fell below 0.9 ± 0.2 mg/L (hypoxic stress), the cover was removed to allow the water to reoxygenate. Six fish were randomly selected at each of the following five time points: normoxia (dissolved oxygen, 7.0 ± 0.2 mg/L), acute hypoxic stress (dissolved oxygen, 0.9 ± 0.2 mg/L), and 4 h, 12 h, and 24 h after the water returned to normoxic levels (dissolved oxygen, 7.0 ± 0.2 mg/L). The fish to be sampled were rapidly anesthetized in MS-222 (300 mg/L), and then the gills were carefully removed. Gill samples from different fish at each timepoint were pooled, transferred to centrifuge tubes (each sample contains tissues from six fish). The gill tissue samples collected in normoxic conditions were labeled as GC (gill tissue normoxia control group), hypoxic samples were labeled as GH (gill tissue hypoxia experimental group), and reoxygenation groups at 4 h, 12 h, and 24 h were labeled as GR_4, GR_12, and GR_24, respectively. Then, the samples were flash frozen in liquid nitrogen, and stored at −80 °C. The experiment was replicated a total of three times.

### 2.5. Metabolite Extraction

Each pooled tissue sample (100 mg) was ground in liquid nitrogen, and the homogenate was resuspended in pre-chilled 80% methanol using a well vortex. After incubation on ice for 5 min, the suspensions were centrifuged at 15,000× *g* and 4 °C for 20 min. Aliquots of each supernatant were diluted in LC–MS grade water to a final concentration of 53% methanol and subsequently transferred to fresh Eppendorf tubes. The diluted samples were centrifuged at 15,000× *g* and 4 °C for 20 min before injection into the LC–MS/MS system. LC–MS/MS was performed as described previously [13].

### 2.6. Data Processing and Metabolite Identification

The raw data files were imported into the CD 3.1 library search software (Thermo Fisher, Waltham, MA, USA) for processing. Metabolites were screened based on parameters such as retention time and mass-to-charge ratio. Then, the retention time deviation was set to 0.2 min and the mass deviation was set to 5 ppm in order to perform peak analysis of the samples. The identification results were aligned to improve accuracy. Next, the mass deviation was set to 5 ppm, the signal intensity deviation was set to 30%, and the signal-to-noise ratio was set to 3. Minimum signal intensity, summing ion, and other parameters were set as appropriate for peak extraction. Peaks were extracted and peak areas were quantified simultaneously. Then, the target ion was integrated. The molecular formulas were predicted based on molecular ion peaks and fragment ions, and these predictions were compared with the mzCloud, mzVault, and Masslist databases. Blank samples were used to remove background ions and normalize the original quantitative results. The recovered metabolites were first identified using the METLIN database (https://metlin.scripps.edu, accessed on 16 October 2021), and taxonomic information for the identified metabolites was obtained using the HMDB database (https://hmdb.ca, accessed on 27 October 2021). The metabolites were then annotated using the KEGG database, and the corresponding KEGG pathways were identified. Metabolites were considered significantly differentially abundant when they had Variable Importance in Projection (VIP) scores >1.0, fold-change (FC) values >1.2 or <0.833, and *p* values < 0.05. Finally, through the above process, the quantitative results of the experimentally relevant metabolites are obtained.

## 3. Results

In positive and negative ion modes, LC–MS/MS analysis identified 821 and 383 metabolites, respectively, in the *T. obscurus* gill. Between acute hypoxia and normoxia, 136 metabolites were differentially abundant in positive ion mode and 34 metabolites were differentially abundant in negative ion mode. The metabolites which were differentially abundant in response to acute hypoxic stress included glycerophospholipids, fatty acids, vitamins, organic acids and their derivatives, heterocyclic compounds, hormones and related compounds, and amino acids.

### 3.1. Data Quality Analyses

**Figure 1:**
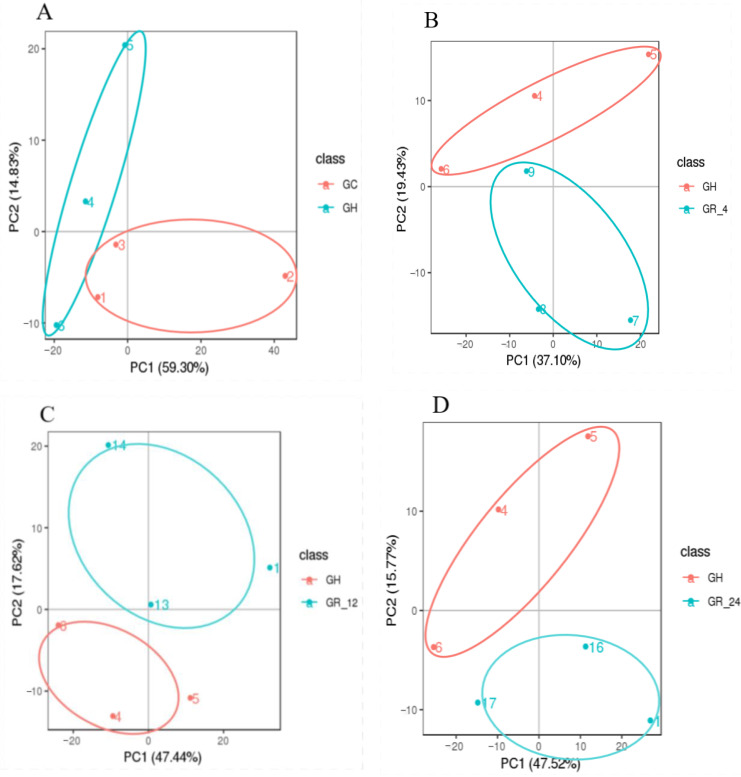
The principal component analysis (PCA) plots of the metabolomic data. (**A**) Groups GH and GC, (**B**) groups GH and GR_4, (**C**) groups GH and GR_12; (**D**) groups GH and GR_24. Each point represents one replicate pooled gill sample, the different numbers represent sample numbers.

PCA (Principal Component Analysis) of a given set of samples may yield a preliminary understanding of the overall metabolic differences among groups of samples, as well as the magnitude of these differences. PCAs of the hypoxic samples (group GH), normoxic samples (group GC), and reoxygenated recovery samples (groups GR_4, GR_12, and GR_24) showed a clear separation between the hypoxic group (GH) and all other groups (Figure 1). The pairwise Pearson’s correlation coefficients between the quality control values for both modes were 0.97 (Supplementary Materials Figure S1), indicating that the MS data had good stability, repeatability, and accuracy. These high correlations also indicated that hypoxia affects relative metabolic abundance in the gill and suggested that this system was suitable for further metabolomic analysis.

**Figure 2:**
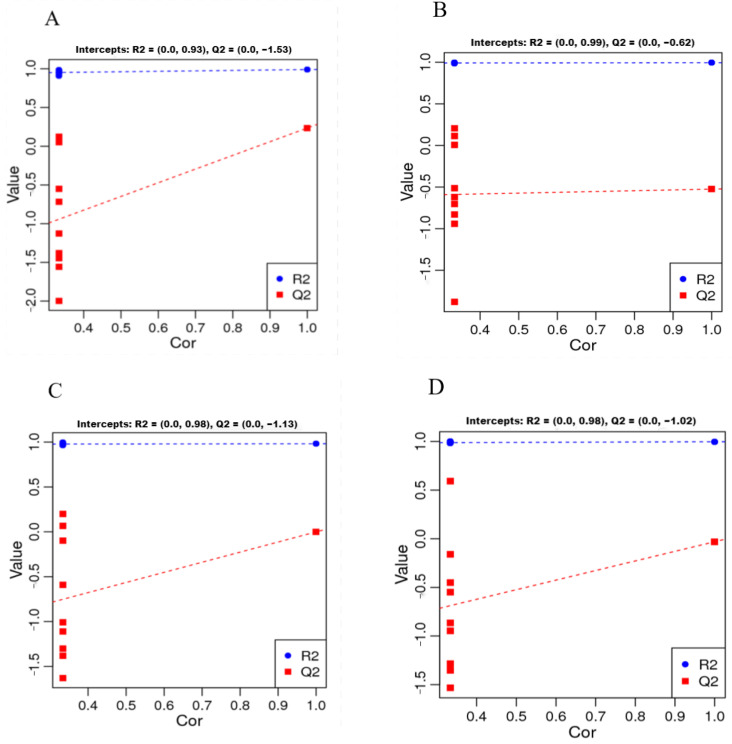
The partial least squares discriminant analysis (PLS-DA) plots of the metabolomic data. (**A**) Groups GH and GC, (**B**) groups GH and GR_4, (**C**) groups GH and GR_12; (**D**) groups GH and GR_24. Each point represents one replicate pooled gill sample.

PLS-DA (partial least squares discriminant analysis) confirmed that the metabolites abundant in the gills of *T. obscurus* in the hypoxic samples (GH) formed a cluster distinct from the normoxic samples (GH) and the reoxygenated recovery samples (GR_4, GR_12, and GR_24). In permutation tests of the PLS-DA results, the R2 metric, which describes the percentage of variation explained by the model, was consistently greater than the Q2 metric, which describes the predictive ability of the model (Figure 2). In addition, the intercept of the Q2 regression line with the Y-axis was less than 0 for all comparisons (Figure 2). These results indicated that the established model was stable and reliable, reflecting significant differences between GH and all other groups (Figure 2). Thus, we deemed our metabolomic data suitable for subsequent data analysis.

### 3.2. Identification of Differentially Abundant Metabolites

In the GC and GH comparison group, we identified 36 differentially abundant metabolites, of which 14 were upregulated in GC as compared to GH, and 22 were downregulated. In the GR_4 and GH comparison group, we identified 18 differentially abundant metabolites, of which 6 were upregulated in GR_4 as compared to GH, and 12 were downregulated. In the GR_12 and GH comparison group, we identified 24 differentially abundant metabolites, of which 4 were upregulated in GR_12 as compared to GH, and 20 were downregulated. In the GR_24 and GH comparison group, we identified 34 differentially abundant metabolites, of which 11 were upregulated in GR_24 as compared to GH, and 23 were downregulated (Table 1).

**Figure 3:**
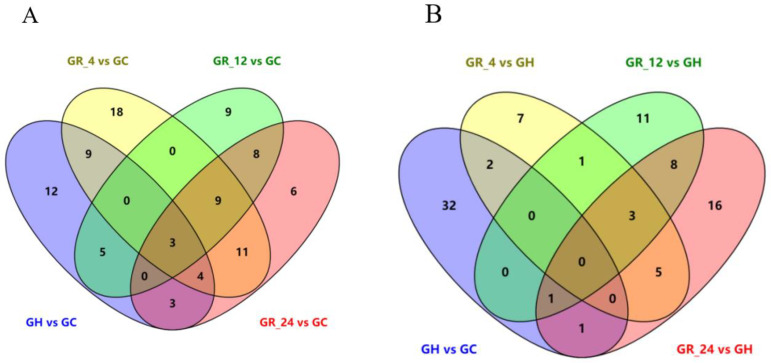
Venn diagrams showing differentially abundant metabolites shared and unique among the hypoxia, normoxia, and recovery groups. (**A**) Differentially abundant metabolites shared and unique across the hypoxia (GH) and recovery (GR_4, GR_12, and GR_24) groups as compared to the normoxia group (GC). Blue indicates GH vs GC group, yellow indicates GR_4 vs GC group, green indicates GR_12 vs GC group, pink indicates GR_24 vs GC group. (**B**) Differentially abundant metabolites shared and unique across the normoxia (GC) and recovery (GR_4, GR_12, and GR_24) groups as compared to the hypoxia group (GH). Blue indicates GH vs GC group, yellow indicates GR_4 vs GH group, green indicates GR_12 vs GH group, pink indicates GR_24 vs GH group. Numbers indicate the number of differentially abundant metabolites.

We drew Venn diagrams to identify the differentially abundant metabolites shared and unique between normoxia and all other groups (Figure 3A) and between hypoxia and all other groups (Figure 3B). Three significantly differentially abundant metabolites (Norbuprenorphine, Stearamide, and 1-Oleoyl-Sn-Glycero-3-Phosphocholine) were shared across the hypoxia and recovery groups as compared to the normoxia group (Figure 3A). Three other significantly differentially abundant metabolites are lipids [PC (plasma cell) (6:0/13:1), PC (19:2/18:5), and PC (20:3/22:6)], and were shared across all three recovery groups as compared to the hypoxia group (Figure 3B). Combining the results of these two figures, it is concluded that most of the metabolites that undergo significant changes during hypoxia and reoxygenation are lipids, followed by amino acids.

Hierarchical clustering analysis identified several groups of metabolites that were differentially abundant between GH and all other groups (Supplementary Materials Figure S2). The metabolite groups with the greatest differences in abundance under hypoxia included the glycerophospholipids (e.g., phosphatidylcholine 6:0/13:1, 4:0/4:0, and 22:4/22:4), fatty acyls (e.g., N-Stearoyl taurine, stearamide, and ACar 12:1), purine nucleosides (e.g., uric acid, adenosine 5’-monophosphate, and deoxyguanosine), steroids (e.g., testosterone), and organic compounds (e.g., indole, lapachol, and norbuprenorphine). Significant, but less dramatic, differences in abundance were observed for organic acids and their derivatives (e.g., indole-3-pyruvic acid) and amino acids (e.g., tyrosine, glutamylcysteine, and citrulline; Supplementary Materials Figure S2).

### 3.3. Metabolic Pathways Responsive to Hypoxic Stress

**Figure 4:**
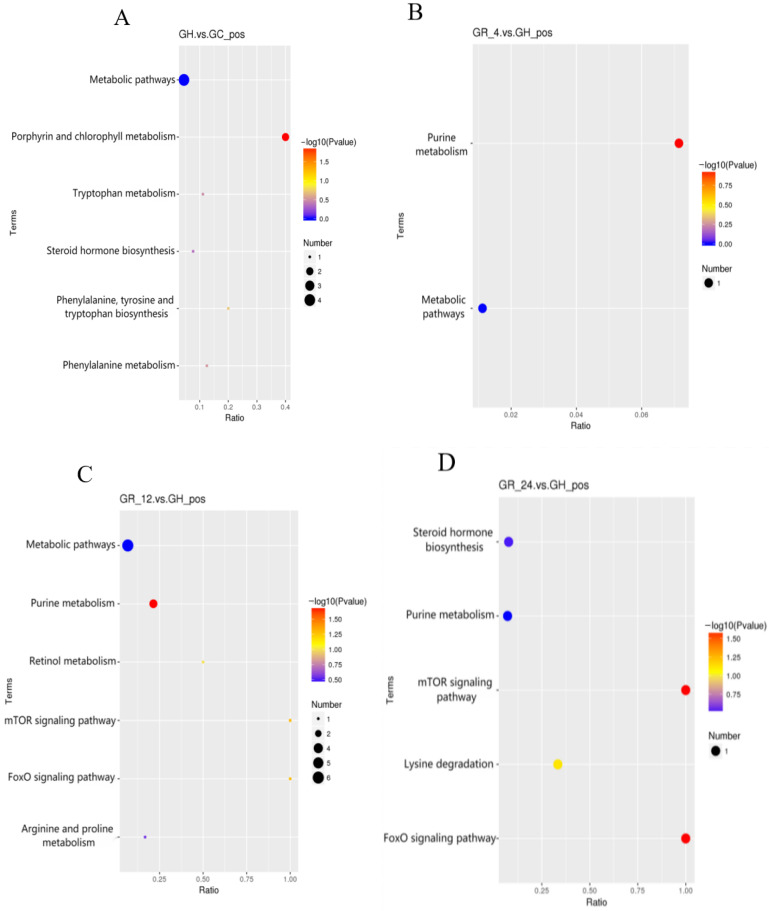
The KEGG pathways most significantly enriched in the metabolites which were differentially abundant between group GH and (**A**) group GC, (**B**) group GR_4, (**C**) group GR_12; and (**D**) group GR_24. In all panels, the Rich factor (the ratio of the number of differentially abundant metabolites in the corresponding pathway to the total number of annotated metabolites) is plotted on the abscissa; higher Rich factors correspond to greater enrichment. Dot color corresponds to significance (deeper reds are more significant), and dot size reflects the number of differentially abundant metabolites in the corresponding pathway.

In order to clarify the functions of the metabolites which were differentially abundant during hypoxia, and to explore the potential metabolic pathways affected by acute hypoxic stress in T. obscurus, we analyzed the KEGG enrichment of the metabolites which were differently abundant in the hypoxic group (GH) as compared to the normoxic groups (GC, GR_4, GR_12, and GR_24). Several KEGG pathways were significantly enriched in the metabolites which were differentially abundant in group GH as compared to each of the other groups (*p* < 0.05; Figure 4). The pathway that differed most significantly between GH and GC was the “porphyrin and chlorophyll metabolism,” containing the metabolites porphobilinogen and picrotoxin, followed by “phenylalanine, tyrosine, and tryptophan biosynthesis,” containing the metabolite indole (Figure 4A). The pathway that differed most significantly between GH and GR_4, as well as between GH and GR_12, was the “purine metabolic pathway,” containing the metabolite uric acid (Figure 4B,C). The pathways that differed most significantly between GH and GR_24 were the “mTOR signaling pathway” and the “FoxO signaling pathway,” both containing the metabolite adenosine-5′-monophosphate (Figure 4D). In total, eight metabolic pathways were significantly enriched in six differentially abundant metabolites (Table 2). Two of these signaling pathways play important regulatory roles in hypoxic stress: the FoxO signaling pathway and the mTOR signaling pathway.

## 4. Discussion

In order to better understand the physiological mechanisms of acute hypoxic stress in *T. obscurus*, we used a metabolomics approach based on LC–MS to comprehensively analyze the hypoxia-induced metabolic changes in the gills. We identified a variety of metabolites that were differentially abundant during hypoxia, including lipids and amino acids. KEGG enrichment analysis suggested that acute hypoxic stress mainly affected the lipid metabolism, amino acid metabolism, purine metabolism, FoxO signaling pathway, and mTOR signaling pathway in the gills. These experimental results provide a scientific basis for future investigations of the metabolic changes in the *T. obscurus* gill associated with the hypoxia response.

### 4.1. Lipid Metabolism

Under hypoxic conditions, organisms must adjust their energy requirements to maintain basic physiological processes [14]. This process often involves the lipid metabolism, as lipids are the main energy source for living organisms [15], and the lipid metabolism is the primary energy production pathway. The lipid metabolism involves a series of complex biochemical reactions, which require sufficient oxygen supply and eventually release large amounts of energy. By comparing the lipid metabolites differentially abundant under normoxia and hypoxia, we found that most of the metabolites that were more abundant during normoxia were less abundant during hypoxia. The metabolites which were downregulated during hypoxia included stearamide, oleamide, testosterone, and palmitoylcarnitine, suggesting that hypoxia inhibits the β-oxidation of fatty acids, causing a decrease in the activity of carnosyl lipid acyltransferase I and inhibiting the entry of long-chain lipid acyls into the mitochondria during fatty acid oxidation [16]. Because hypoxia inhibits normal gill functions, including respiration, CO_2_ and H_2_O levels decrease due to the reduction in oxidative decomposition, leading to substantial decreases in the energy obtained by the organism. Acute hypoxic stress also inhibits adipocyte differentiation and production and decreases the rate of fatty acid oxidation and uptake, which may inhibit aerobic metabolic processes and lead to a negative energy balance [17]. Under hypoxia, the energy balance depends on anaerobic glycolysis [18], as was previously demonstrated in the skeletal muscles of rats under hypoxia [19]. In addition, it was found that goldfish hearts also undergo anaerobic glycolysis in vivo to mitigate the negative consequences of hypoxia-dependent ATP production [20]. It was also found that the livers of puffer fish under hypoxic stress also require pyruvate produced by glycolysis for energy metabolism. Most importantly, the conversion from aerobic to anaerobic metabolism is a key adaptation mechanism for hypoxia tolerance in fish [12].

Our results also showed that glycerophospholipid metabolites were significantly more abundant under hypoxia as compared to normoxia and reoxygenation. As the most abundant phospholipids in the body, glycerolphospholipids not only constitute biofilms, but also participate in protein recognition and signal transduction by cell membranes. Under acute hypoxic stress, the structure of the cell membrane is maintained by increasing the metabolism of glycerolphospholipids [21], suggesting that *T. obscurus* may produce more glycerolphospholipids to help maintain the stability of the cell membrane under hypoxic stress. In addition, some phosphatidylcholines, such as PC (3:0/13:1), PC (4:0/4:0), PC (6:0/13:1), and 1-Oleoyl-Sn-Glycero-3-Phosphocholine, are produced when the body is deprived of oxygen. Phosphatidylcholine is an osmolyte that can counteract the effects of urea on enzymes and other macromolecules [22], which helps to alleviate the damage associated with the accumulation of nitrogen metabolic wastes that cannot be excreted normally. The significant increase in phosphatidylcholine metabolites in *T. obscurus* under hypoxic stress may reflect this detoxification function.

Finally, our experimental results showed that the abundance of steroid metabolites (e.g., testosterone, methyltestosterone) decreased significantly in response to hypoxic stress in *T. obscurus*, and this decrease was irreversible, with steroid metabolites remaining at low abundance even after 24 h of reoxygenation. Sterol biosynthesis may be permanently disrupted after hypoxic stress, because hypoxia disrupts the oxygenation step of sterol biosynthesis and interferes with the intracellular cholesterol metabolism [23]. Thus, the downregulation of sterol metabolites during hypoxia in *T. obscurus* can affect the regulation of cell membrane function, as well as the salt and water balance.

Together, our results indicated that lipid-related metabolites respond strongly to hypoxia in order to maintain normal gill function in *T. obscurus*.

### 4.2. Catabolism of Amino Acids

The results showed that the biosynthesis of phenylalanine, tyrosine, and tryptophan, as well as the metabolism of phenylalanine, played an important role in hypoxic stress. In many organisms, phenylalanine, tyrosine, and tryptophan biosynthesis increases after hypoxic stress to ensure a normal supply of these amino acids, which are required for energy synthesis. In particular, the abundance of L-phenylalanine (a phenylalanine derivative) increased significantly in the *T. obscurus* gill during hypoxia. At high concentrations, phenylalanine acts as a neurotoxin and damages nerve cells [24]. Thus, *T. obscurus* may have sustained nerve damage in the gill under hypoxia. Indeed, a previous study of sodium bicarbonate stress in Founder’s silver carp [25] also showed that environmental changes can stimulate the phenylalanine metabolism, increasing phenylalanine content in the gill and leading to nerve damage. Tyrosine, tryptophan, and valine were significantly more abundant in the *T. obscurus* gill during hypoxia as compared to normoxia and reoxygenation. Because these amino acids are important for fish metabolism, growth, and development, we speculated that the biosynthesis of these amino acids was temporarily increased to offset hypoxia-associated disruption of gill function in *T. obscurus*.

### 4.3. Purine Metabolism

The end product of the purine metabolism is uric acid, which is an important antioxidant that helps to maintain stable blood pressure and to reduce oxidative stress [26]. Uric acid was significantly more abundant under hypoxic conditions as compared to normoxia and reoxygenation, suggesting that hypoxia disrupted normal gill function, destabilized blood pressure, and increased oxidative stress. These results were consistent with a previous study of oxidative damage associated with hypoxia in carp [27]. In addition, it has been shown that, under hypoxic conditions, ATP in fish tissues is converted to adenosine and xanthine, depleting overall levels of ATP, which reduces the amount of energy available to the body. After reoxygenation, the accumulated xanthine is converted to uric acid by the enzyme xanthine oxidase. As a by-product of this reaction, the mitochondria release a variety of reactive oxygen species, such as superoxide anions and peroxyl radicals [28]. These reactive oxygen species can induce oxidative stress and lead to lipid peroxidation, resulting in tissue damage and even apoptosis [29]. During reoxygenation, the antioxidant system is triggered to counteract the damage caused by oxidative stress [30]. Thus, the significant increase in adenosine uric acid content in response to hypoxia in the *T. obscurus* gill may reflect tissue damage.

### 4.4. Hypoxia-related Signaling Pathways

#### 4.4.1. The FoxO Signaling Pathway

**Figure 5:**
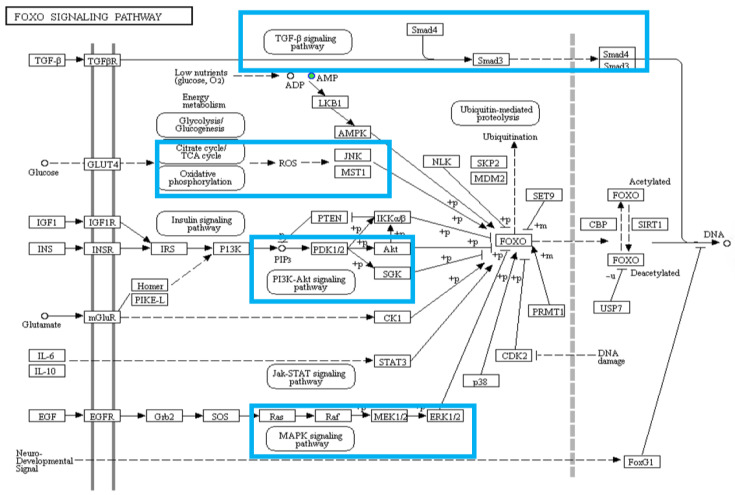
Schematic showing the FoxO signaling pathway under hypoxic stress. The main processes affected by hypoxia are boxed in blue.

The results showed that the FoxO signaling pathway played a role in the response to hypoxia. The FoxO protein plays a critically important role in the cellular oxidative stress response. FoxO is activated in response to oxidative stress, energy deficiency, and DNA damage via a number of processes, including phosphorylation, monoubiquitination, methylation, and glycosylation (Figure 5). In addition, recent studies have suggested that FoxO regulates genes participating in a number of functions, including the cell cycle, cell death, cell autophagy, cell metabolism, and cellular antioxidation [31]. It also plays an important role in maintaining reactive oxygen species homeostasis [32]. In a hypoxic environment, FoxO responds to changes in the cellular redox state by altering the upstream FoxO regulatory pathway or by the reversible oxidation and reduction in cysteine residues to ameliorate oxidative stress [33]. Hypoxic stress activates the transforming growth factor pathway (TGF-β) via the signal transduction factors Smad3 and Smad4, resulting in DNA damage and leading to apoptosis. Hypoxia also leads to decreases in ATP production and increases in reactive oxygen species content. Adenosine 5‘-monophosphate (AMP)-activated protein kinase (AMPK), which functions as an energy sensor for metabolic adaptations under ATP-deprived conditions such as hypoxia, is activated by increases in adenosine monophosphate or reactive oxygen species. The excessive production of reactive oxygen species activates the protein kinase JNK, and the activated JNK can induce apoptosis [34]. JNK translocation into the nucleus can regulate the activity of various transcription factors, promote the expression of FoxO, and reduce DNA damage. FoxO expression is also affected by the phosphatidylinositol kinase (P13K)-Akt signaling pathway. P13K activates Akt by changing its protein structure, while pyruvate dehydrogenase kinase 1 (PDK1), an upstream activator of Akt, phosphorylates Akt at the threonine 308 site. This leads to the partial activation of Akt and thus regulates cell proliferation, differentiation, and apoptosis [35]. MAPKs, a family of enzymes involved in oxygen sensing, are activated under hypoxia and play a regulatory role in a variety of important pathophysiological processes, such as stress responses and adaptations to environmental changes; MAPKs influence FoxO expression as well [36]. A previous study showed that p44 MAPK (ERK) in hypodermal fibroblasts was involved in the hypoxia adaptation of rainbow trout [37].

FoxO1, a member of the FoxO family, activates anti-apoptotic genes and protects gill cells from oxidative stress damage. Similarly, in fish, endogenous FoxO1 was shown to improve resistance to oxidative stress and exogenous FoxO1 was shown to upregulate endogenous FoxO1 [38]. In addition, under oxidative stress, FoxO1 can initiate upstream or downstream genes to resist oxidative stress damage leading to disease development [39]. Thus, accumulation of FoxO in the *T. obscurus* gill in response to hypoxia, as well as the significant enrichment of the FoxO pathway in the differentially abundant metabolites, suggested that this pathway is critical to the adaptation of *T. obscurus* to acute hypoxia, helping to reduce apoptosis and to protect body tissues from damage.

#### 4.4.2. The mTOR Signaling Pathway

**Figure 6:**
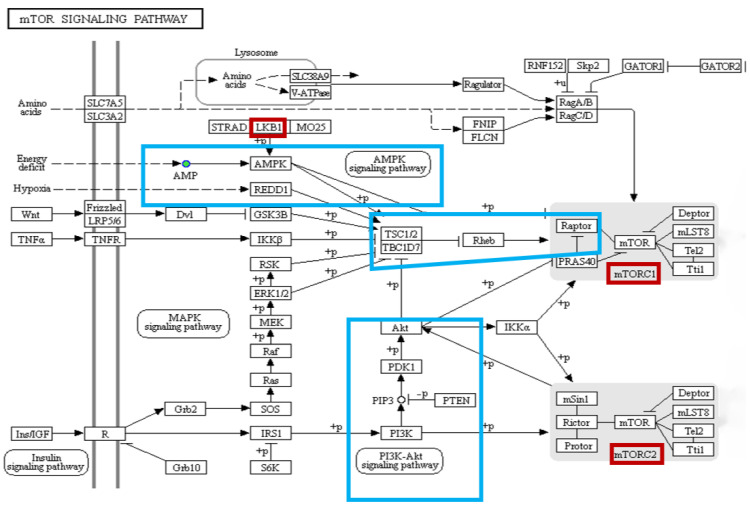
Schematic showing the mTOR signaling pathway under hypoxic stress. The main processes affected by hypoxia are boxed in blue, and the major regulatory factors are boxed in red.

The mTOR signaling pathway may also play an important role in hypoxia adaptation [40]. The mTOR protein complex is activated in response to hypoxic stress, low energy production, and nutrient deprivation (Figure 6). AMP, an important metabolite in the mTOR signaling pathway, was significantly more abundant under hypoxia in the gill of *T. obscurus.* This was consistent with the significant increase in AMP in response to hypoxia in darkling oriental triggerfish, as well as the upregulation of AMP in response to hypoxia in goldfish [41] and rainbow trout [42]. Under hypoxia, AMP activates AMPK, a major regulator of the energy metabolism, in conjunction with LKB1, an upstream kinase of AMPK [43]. In a hypoxic environment, AMPK restores the energy balance by activating catabolic pathways; regulating glucose uptake, glycolysis, and fatty acid oxidation; and promoting ATP production to replenish some of the energy lost during acute hypoxic stress.

Together, LKB1 and AMPK activate most members of the AMPK-related protein kinase superfamily [43]. Once AMPK is activated, energy depletion leads to the tuberous sclerosis complex (TSC1/TSC2)-mediated inhibition of mTOR. As shown in Figure 6, LKB1 is regulated by AMPK phosphorylation and can directly phosphorylate the TSC1/TSC2 complex, resulting in the conversion of Rheb to the inhibitory form bound to GDP. Blocking of the mTOR pathway then occurs, thereby inhibiting tumor growth and cellular metabolic activity. Meanwhile, the REDD1 gene acts as a repressor of mTOR activity, inhibiting mTOR when cells are stimulated by hypoxia, a process that is dependent on the presence of the functional complex TSC1/TSC2 [44,45]. Notably, AMPK is highly sensitive to elevated intracellular AMP/ATP ratios and is therefore a key energy-sensitive kinase. Here, AMP was upregulated during hypoxia in *T. obscurus*, increasing the intracellular AMP/ATP ratio and thus activating AMPK. The activated AMPK phosphorylated TSC2, which, in turn, inhibited the activity of mTOR [46]. In addition, mTOR can sense and integrate environmental signals through two protein complexes, mTORC1 and mTORC2 [47]. In response to the insulin signaling PI3K-AKT-mTOR pathway, AKT directly promotes glucose uptake while activating mTORC1 activity via AKT-TSC1/2-Rheb-mTORC1 [48]. mTORC1 directs the synthesis of enzymes related to biosynthesis using glucose for nutrient storage, and promotes the absorption of nutrients by gill cells. mTORC1 further actively regulates cellular metabolism to produce ATP in order to ensure energy supply under hypoxic conditions.

## 5. Conclusions

In this study, we used LC–MS to quantify changes in the abundance of metabolites in the gills of *T. obscurus* in response to acute hypoxia stress. We found that the pathways most enriched in the differentially abundant metabolites included the amino acid metabolism, purine metabolism, FoxO signaling pathway, and mTOR signaling pathway. Based on the physiological functions of these pathways and the associated differentially abundant metabolites, our results suggested that hypoxic stress affects *T. obscurus* immunity, energy acquisition, and anti-apoptotic ability. This study helps to clarify the mechanisms of hypoxia tolerance in fish, to identify hypoxia-related metabolites, and to highlight possible molecular research targets that may support the cultivation of hypoxia-tolerant fish strains in the future.

## Figures and Tables

**Table 1:** Differentially abundant metabolites between groups.

Comparison	Differentially Abundant Metabolites	Metabolites More Abundant in GH
GC vs. GH	36	22
GR_4 vs. GH	18	12
GR_12 vs. GH	24	20
GR_24 vs. GH	34	23

**Table 2:** Metabolites which were significantly differentially abundant in the gills of *Takifugu obscurus* under hypoxic stress and the associated metabolic pathways.

Metabolite	VIP	FC	P	Trend	Metabolic pathway
Porphobilinogen	1.61	2.92	0.049	↑	Porphyrin and chlorophyll metabolism
Indole	1.63	1.78	0.022	↑	Phenylalanine, tyrosine and tryptophan biosynthesis Tryptophan metabolism
2-Phenylacetamide	1.59	1.76	0.027	↑	Phenylalanine metabolism
Testosterone	1.67	0.21	0.047	↑	Steroid hormone biosynthesis
Uric acid	1.62	0.67	0.036	↑	Purine metabolism
Adenosine 5′-monophosphate	1.73	0.57	0.033	↑	FoxO signaling pathway mTOR signaling pathway

Notes: ↑ upregulation, VIP: Variable Importance in Projection, FC: Fold Change, P: *p* value.

## Data Availability

The data presented in this study are available in this article and supplementary material.

## Supplementary Materials

The following supporting information can be downloaded at: https://www.mdpi.com/article/10.3390/ani12192611/s1, Figure S1: Pairwise correlations between QCs in (A) positive and (B) negative ion mode. Figure S2: Hierarchical clustering of differentially abundant metabolites.
