# Supplementary material for: Metabolomic Analysis of the Takifugu Obscurus Gill under Acute Hypoxic Stress
Source: Animals (Basel). 2022 Sep 29;12(19):2611. doi: 10.3390/ani12192611 (PMC9559691; doi:10.3390/ani12192611)
Supplement: Supplementary file 1 [file animals-12-02611-s001.zip › animals-1916517-supplementary.pdf]

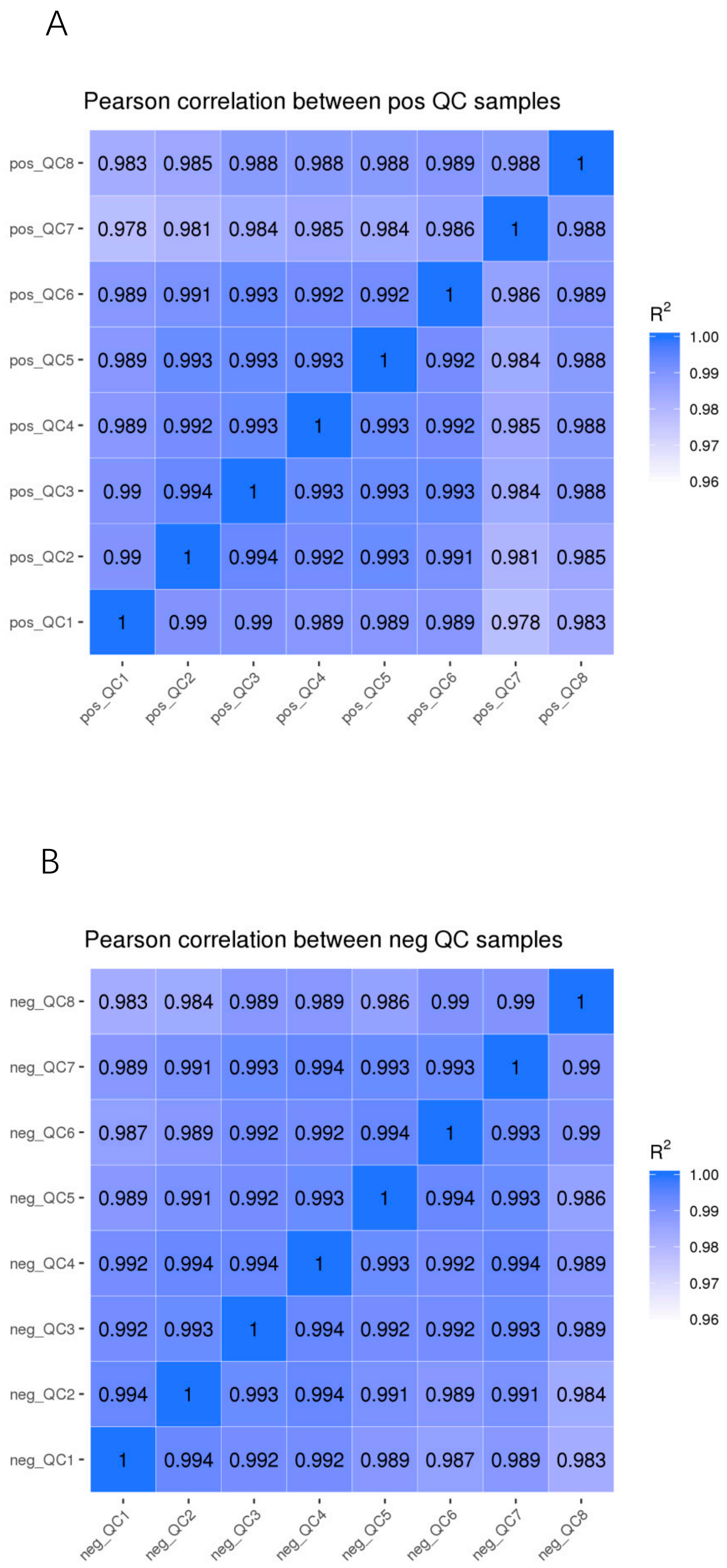

Figure S1. Pairwise correlations between QCs in (A) positive and (B) negative ion mode.

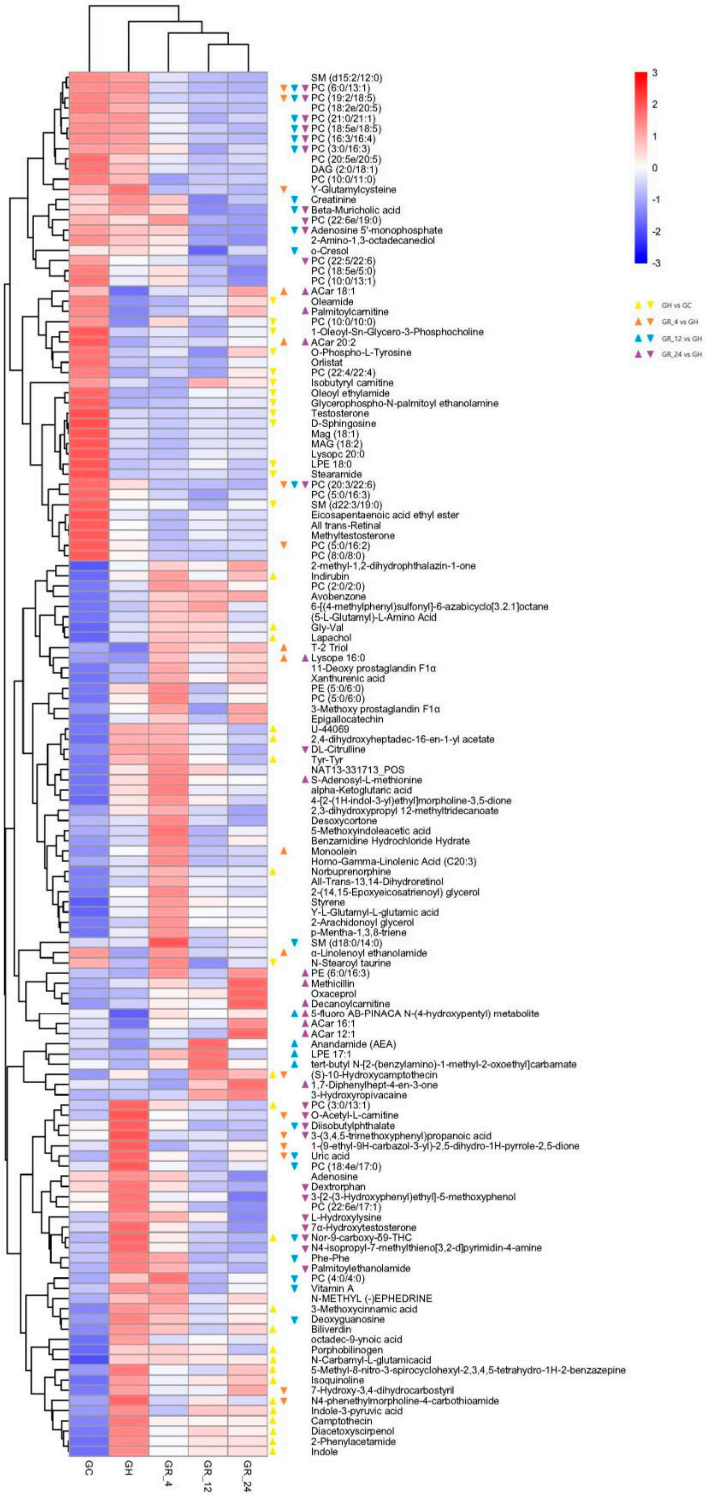

Figure S2. Hierarchical clustering of differentially abundant metabolites. Red cells correspond to more abundant metabolites and blue cells correspond to less abundant metabolites. Upward-pointing triangles indicate that the corresponding metabolite was more abundant in the comparison group, while downward-pointing triangles indicate that the corresponding metabolite was less abundant in the comparison group.
